# Enhancing energy autonomy of greenhouses with semi-transparent photovoltaic systems through a comparative study of battery storage systems

**DOI:** 10.1038/s41598-025-85418-z

**Published:** 2025-01-17

**Authors:** Mohammadreza Gholami, Ali Arefi, Anwarul Hasan, Chengdao Li, S. M. Muyeen

**Affiliations:** 1 Department of Electrical and Electronic Engineering, Final International University, Kyrenia, 99320 Turkey; 2 School of Engineering and Energy, Murdoch University, Perth, Australia; 3 Mechanical and Industrial Engineering, Qatar University, 2713 Doha, Qatar; 4 Western Crop Genetics Alliance, Food Futures Institute, School of Agriculture, Murdoch University, Perth, WA Australia; 5 Department of Electrical Engineering, Qatar University, 2713 Doha, Qatar

**Keywords:** Battery energy storage system, Daily lighting integral, Greenhouse, Semi transparent photovoltaic, Harmony search (HS), Electrical and electronic engineering, Environmental economics, Sustainability

## Abstract

Effective energy management is crucial in greenhouse farming to ensure efficient operations and optimal crop growth. This study investigates the energy autonomy—defined as the ratio of on-site energy generation to the total energy demand—of greenhouses equipped with semi-transparent photovoltaic (STPV) systems under two scenarios: with and without a Battery Energy Storage System (BESS). STPV systems are beneficial because they generate energy while still allowing enough light to pass through for healthy plant development. Seasonal variations in energy autonomy during summer and winter were analyzed. Results show that incorporating BESS significantly reduces reliance on grid electricity, with energy autonomy improving from 43.43% to 24.17% in summer and 81.36% to 69.45% in winter. The system’s performance was highly sensitive to the transmittance rate of STPV panels and the minimum Daily Light Integral (DLI) required for crops. These findings highlight the potential of BESS to enhance energy independence and promote sustainable agricultural practices. The study provides insights into optimizing renewable energy systems in greenhouses, emphasizing practical implications for scalability and economic feasibility.

## Introduction

Greenhouse technology plays an essential role in modern agriculture by enabling the controlled environment cultivation of a wide variety of crops. This controlled environment ensures optimal growing conditions, independent of external weather patterns, which leads to higher yields and improved crop quality. The importance of greenhouses is underscored by their significant contribution to food security, particularly in regions with less favorable growing conditions. The Food and Agriculture Organization emphasizes the critical role of greenhouse technology in meeting the projected 70% increase in global food production demand^1^. Currently, greenhouse agriculture spans over millions of hectares of lands worldwide, with a substantial presence in eastern Asia and the Mediterranean regions^2^. While the adoption of greenhouse technology significantly enhances food production capabilities, it also leads to increased energy consumption^3^. As a result, this contributes to higher greenhouse gas emissions and a greater carbon footprint^4^. In this context, integrating renewable energy sources (RES) into greenhouse operations is not only going to be beneficial but necessary. Photovoltaic (PV) technology, with its decreasing costs, stands out as a promising solution. European union, for instance, is planning a significant increase in PV capacity, aiming to exceed 200 GW over the next decade^5^.

### Semi-transparent photovoltaic systems

**Fig. 1:**
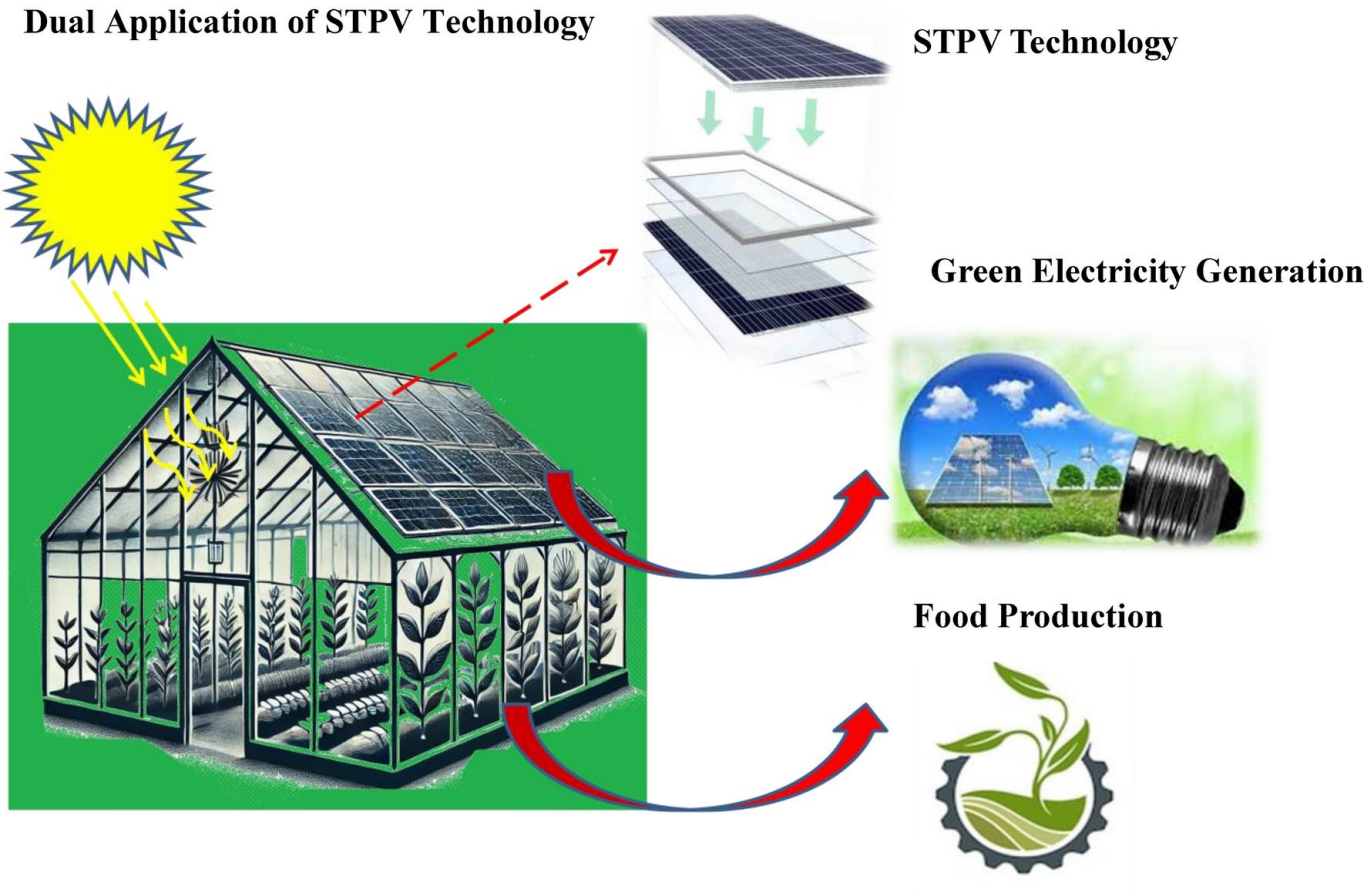
Dual application of STPV in a greenhouse.

Agricultural photovoltaic, which combine PV power generation with traditional farming practices, presents a synergistic approach^6^. This approach addresses the challenges of energy demand in agriculture. Additionally, it contributes to sustainable farming practices by reducing dependence on non-renewable energy sources^7^. By installing PV systems on croplands, which are rich in solar resources, greenhouses are able to lower their dependency on fossil fuels. Integrating Semi-transparent photovoltaic (STPV) systems into greenhouses further enhances this synergy by allowing sufficient light for plant growth while simultaneously generating electricity (Fig. 1). STPV systems represent an innovative approach for integrating solar energy generation with light transmission, making them particularly suitable for applications such as greenhouses^8^. This dual functionality not only helps in maximizing land use efficiency but also aligns with sustainable agricultural practices. Unlike traditional opaque PV panels, STPV systems are designed to allow a portion of sunlight to pass through while converting the rest into electricity^9^. This selective light transmission is crucial for maintaining optimal photosynthesis conditions. Additionally, this dual functionality is achieved through various technologies^10^. Furthermore, STPV technology can contribute to enhanced climate resilience in agricultural practices. The ability to produce energy on-site reduces the carbon footprint associated with energy transportation and infrastructure. Compared to traditional PV systems, STPV offers the unique advantage of simultaneous energy generation and light transmittance, which is crucial for maintaining the Daily Light Integral (DLI) required for crops. Wind energy, while effective in some regions, is less reliable and difficult to integrate within the structural design of greenhouses. The dual functionality of STPV systems, combined with the flexibility of BESS, positions this approach as a superior solution for achieving energy autonomy in greenhouse farming.

Table 1, presents information on various types of semi-transparent photovoltaic technologies, their materials, efficiencies, transparency levels, and additional relevant notes. STPV systems can employ different methods to balance light transmission and energy conversion, such as using different types of materials or designing varying layers within the panel.

**Table 1:** Summary of semi-transparent photovoltaic technologies.

Technology	Material	Efficiency (%)	Transparency (%)	Note
Thin Film	Titanium dioxide (TiO2), Nanocrystalline materials	7.8–9.2	20–60	Offers a balance between efficiency and transparency, but transparency can vary significantly based on material choice and thickness
Near-Infrared	C60, TiO2, SiO2, …	1.2–1.4	> 65	Provides high transparency by targeting UV and NIR absorption, though efficiency remains low
Polymer Solar Cell	Low band gap polymers, PCBM, …	4.0–7.6	25–68	Allows visible light transmission, with moderate efficiency, but transparency depends on material selection
Transparent Luminescent Solar Concentrator	Organic salts	0.4	86	Extremely high transparency but very low efficiency, making it ideal for aesthetics rather than energy production
Perovskite Solar Cell	TiO2, Al2O3, …	6.4–12.7	22–77	Provides a good compromise between efficiency and transparency, though stability and scalability remain concerns
Electrophoretic Deposition	TiO2 nanotubes, FTO glass, polyaniline,…	3.88–8.22	22–70	Promises both high efficiency and transparency, but requires further validation for widespread use

While photovoltaic STPV systems offer significant advantages in renewable energy generation, they are not without their shortcomings. A notable issue is the trade-off between transparency and efficiency, where increasing transparency often results in decreased energy conversion efficiency. STPV systems typically exhibit lower efficiency compared to traditional PV panels, which can lead to intermittent energy production^11^. Since solar panels generate electricity only during daylight hours, their output varies based on weather conditions, time of day, and seasonal changes. This intermittency can pose particular challenges for energy-intensive operations like greenhouses, where a stable energy supply is crucial for maintaining consistent environmental conditions. Periods of low or no power generation, especially during cloudy days or at night, can lead to reliability issues. Additionally, while the efficiency of STPV systems is improving, it remains lower compared to traditional opaque PV panels, meaning they generate less electricity overall^8^. This reduced efficiency can affect the viability of STPV systems as a sole energy source for high-demand applications. Despite these challenges, the lower energy yield of STPV systems might necessitate the use of supplementary energy sources or storage solutions to ensure a reliable power supply. To address these issues, ongoing research and development are focused on enhancing the efficiency and durability of STPV technologies. Future advancements could make STPV systems more viable for widespread use in agricultural settings. As STPV technologies evolve, their integration into greenhouse systems could lead to significant improvements in sustainable agriculture and energy management.

### Optimizing battery storage for greenhouses

Battery Energy Storage Systems (BESS) offer a practical solution to the mentioned shortcomings by storing excess power produced at peak sunlight hours and use it during hours when solar power generation is insufficient^12^. By providing a buffer against the variability of solar power, BESS ensures a reliable and continuous energy supply, which is crucial for greenhouse operations that depend on stable environmental conditions for crop production. In greenhouses, maintaining optimal temperature, humidity, and lighting conditions is vital for plant growth, and any disruptions in power supply can jeopardize these conditions. BESS also helps in load balancing, smoothing out the fluctuations in energy availability and demand^11^. This reduces the greenhouse’s dependency on the grid and can significantly decrease energy costs^13^. By mitigating demand spikes and supplying energy during off-peak periods, BESS can also play a role in reducing the need for expensive grid upgrades, which would otherwise be necessary to handle increased loads. Moreover, by enabling the use of stored renewable energy instead of fossil fuel-based backup generators, BESS contributes to reducing the carbon footprint of greenhouse operations, promoting more sustainable agricultural practices^14^.

The integration of BESS into microgrids energy systems not only supports sustainability goals but also enhances energy security^15^. In the context of agricultural operations, especially those in remote or off-grid locations, BESS provides a critical backup power source, ensuring that vital systems remain operational during power outages or in the absence of sufficient sunlight. This capability is particularly important as climate change leads to more frequent and severe weather events, which can disrupt both solar power generation and grid reliability. In addition, the use of BESS can improve the economic viability of greenhouses by providing a more predictable energy cost structure and reducing the financial risks associated with energy price volatility. Properly sizing BESS is crucial for maximizing their effectiveness in supporting renewable energy systems like STPV in greenhouse operations. The size of BESS determines its capacity to store and discharge energy, directly influencing the system’s ability to meet energy demands during periods of low solar input or high consumption^16^. An undersized BESS may not provide sufficient backup power during extended periods of low solar energy generation, while an oversized system can lead to unnecessary capital expenditure and underutilization. Various methods, including mathematical modeling and optimization techniques, are employed to determine the optimal BESS size and configuration^17^. In^18^, six different optimization algorithms are employed to find the optimum performance of a hybrid battery-supercapacitor energy system. These methods consider factors such as the greenhouse’s energy demand profile, the solar generation potential, weather patterns, and the cost of energy storage technologies^19^. Metaheuristic algorithms have proven effective in handling the complex, nonlinear nature of optimization problems associated with BESS sizing^20,21^. Different heuristic and evolutionary algorithms are investigated in handling the microgrids optimization based problems in^22,23^.

Harmony Search (HS), a metaheuristic algorithm inspired by musicians’ improvisation process, has gained attention for its ability to find near-optimal solutions by iteratively adjusting solutions based on harmony memory and pitch adjustment^24^. The HS algorithm is particularly suited for optimizing energy storage systems because it can efficiently navigate large, multidimensional solution spaces to identify configurations that balance cost, performance, and reliability. In our study, we utilized the Harmony Search algorithm to optimize the size and the spatial distribution of BESS within the greenhouse system^25^. This optimization strategy ensures that energy storage is strategically located to minimize transmission losses and enhance overall system efficiency. By placing storage units closer to high-demand areas, we can reduce energy transmission distances and improve the overall responsiveness of the energy supply system.

The results from our optimization model demonstrate that strategic placement and sizing of BESS can lead to significant improvements in energy efficiency and cost savings. This approach not only enhances the sustainability of greenhouse operations by minimizing energy waste but also contributes to better economic outcomes through reduced operational costs and improved crop yields due to stable environmental conditions. Future research could explore integrating advanced predictive models that incorporate real-time weather data and machine learning algorithms to further enhance the accuracy and effectiveness of BESS optimization in greenhouse environments. As the cost of BESS technology continues to decline and its performance improves, its application in agriculture and other energy-intensive sectors is likely to expand, driving further advancements in renewable energy integration and sustainability.

### Integrating daily light integral in greenhouse energy solutions

The Daily Light Integral (DLI) serves as a critical parameter in greenhouse agriculture, representing the total amount of photosynthetically active radiation (PAR) received by crops over a 24-h period. DLI directly influences plant growth, development, and overall productivity, making it essential to maintain optimal light levels within greenhouses^26^. The amount of PAR a plant receives affects various physiological processes, such as photosynthesis, transpiration, and nutrient uptake, which are all crucial for healthy plant growth. Managing DLI constraints effectively is pivotal in achieving a balanced integration of renewable energy technologies in greenhouse environments, aligning energy efficiency goals with agricultural productivity^27^. Ensuring that the DLI requirements are met allows for maximum plant health and yield, even as renewable energy systems like STPV panels and BESS are integrated into the greenhouse design. Different crops have varying DLI requirements depending on their growth stages and light sensitivity. For instance, high-light crops like tomatoes and peppers require a significantly higher DLI compared to shade-tolerant crops such as leafy greens. Understanding these specific light requirements is essential for tailoring greenhouse conditions to optimize plant health and yield. In our study, we integrated DLI as a primary constraint in our optimization framework for energy management. By considering DLI requirements specific to different crop types, we ensured that energy solutions, including the deployment and operation of STPV systems and BESS, complemented rather than compromised plant growth. This approach enables greenhouse operators to balance energy savings with the provision of adequate light for photosynthesis, thereby supporting sustainable agricultural practices.

In this study, we assume that the crop-specific DLI thresholds are fixed based on existing agricultural guidelines. The DLI values used in the optimization model are based on average lighting needs for high, medium, and low light-demanding crops. While actual DLI requirements may vary due to factors like plant age and health, using established thresholds ensures the results are applicable to a wide range of greenhouse operations.

Moreover, integrating DLI into the energy management strategy helps in reducing the overall energy footprint of greenhouse operations. When DLI is properly managed, it allows for strategic use of supplemental lighting only when natural sunlight is insufficient, thereby minimizing energy consumption. This targeted approach not only optimizes energy use but also enhances crop yield and quality by providing consistent, optimal light conditions. By aligning the DLI management with renewable energy generation patterns, greenhouses can achieve a more sustainable balance between energy consumption and production. Table 2 provides the minimum DLI values for different types of crops. These values are integral to our analysis as they ensure that the integration of STPV panels does not compromise the necessary light conditions for crop cultivation. Incorporating these values into our optimization model allows for a more precise and effective deployment of renewable energy technologies. For example, STPV panels can be strategically placed or designed to ensure they provide sufficient light transmission while also generating energy. This consideration is crucial for high-light crops, where any reduction in PAR due to PV shading could adversely affect growth and yield.

**Table 2:** Minimum DLI for various crops^26,27^.

Crop type	Minimum DLI (mol/m^2^ d)
High light-demanding (e.g., Tomato, Cucumber, Pepper)	30
Medium light-demanding (e.g., Asparagus, Chrysanthemum, Rose, Lettuce, Basil)	10–20
Low light-demanding (e.g., Dracaena, Poinsettia, Kalanchoe)	Less than 10

While the DLI concept plays a critical role in optimizing STPV-BESS systems, several challenges need to be considered. Firstly, accurately determining the required DLI for different crops across varying environmental conditions can be complex. Additionally, ensuring that DLI constraints are effectively integrated into the optimization model requires careful balancing of energy production with crop-specific light needs. Seasonal variations further exacerbate this challenge, as fluctuating weather conditions can impact the accuracy of DLI predictions and necessitate dynamic adjustments to the system. This study addresses these challenges by incorporating a flexible optimization approach that adapts to seasonal changes and ensures optimal crop growth alongside energy management.

### Contribution of the study

While several renewable energy technologies have been proposed for greenhouses, including wind turbines and traditional PV systems, these solutions often lack the dual functionality required for greenhouse environments. Traditional PV systems, for instance, block a substantial portion of sunlight, which can adversely affect crop growth. Additionally, these technologies do not account for the critical agricultural parameters, such as the DLI and specific crop requirements, which directly influence both energy demand and plant productivity. This study aims to investigate how STPV systems, which enable simultaneous energy generation and optimal light transmittance, can be effectively integrated with BESS to improve energy autonomy in greenhouses. By considering the interplay between energy management and agricultural needs—including DLI thresholds and crop types—this research offers a comprehensive approach to optimizing renewable energy systems for sustainable and efficient greenhouse operations.

Previous researches focused on utilizing STPV in a greenhouse. Ref^28^ explores the potential of STPV cladding on greenhouse roofs to generate solar electricity while supporting crop production. The study uses energy and life cycle cost analysis, considering current and future efficiency projections for PV and horticultural lighting technologies. Results indicate that while STPV cladding currently increases lighting electricity use, it could potentially supply the greenhouse’s demand. The internal shading caused by STPV necessitates increased supplemental lighting but reduces heating energy use. Despite its current economic unattractiveness, STPV is expected to become a viable and promising cladding alternative, enhancing energy efficiency and economic performance as technology advances. However, they did not consider the different aspect of using STPV such as decreasing the energy autonomy or increasing the resiliency. Also, they did not consider the effect of energy storage system in their analysis. Ref^29^ investigates the implementation of a new STPV module prototype in a real greenhouse setting. Their proposed technology mitigates shadow effects on crops by ensuring that the cells’ shadows do not completely eclipse the sunlight, promoting a better distribution of solar radiation within the greenhouse. Despite a slight increase in yield ratio due to ground-reflected radiation utilization, the energy produced is still insufficient to meet the greenhouse’s electrical needs. Ref^26^ highlights the significant energy production potential of PV and STPV systems, demonstrating their capability to meet up to 30% of the annual electricity demand. The findings emphasize the importance of the proportion of the projected PV area to the total greenhouse area. In their study, they formulated the average daily radiation inside and outside of the greenhouse considering both PV and STPV panels. Also, they provided comprehensive information about DLI and transparency of STPV.

Above studies did not sufficiently consider the integration of energy storage systems, which is crucial for optimizing energy management in greenhouse environments. Without incorporating energy storage systems such as BESS, the ability to efficiently manage and balance energy supply and demand in real-time is limited. These systems play a vital role in mitigating the intermittent nature of renewable energy sources, particularly in a dynamic environment like greenhouses where energy demand fluctuates with crop growth cycles and seasonal changes. Additionally, while many studies explore the STPV systems to meet greenhouse energy demands, they often fail to provide a clear framework or specific factors that quantify the percentage of total energy demand that can be met with and without energy storage systems. This is essential for assessing the true value of energy autonomy and for understanding how much of the energy demand can be sustainably covered by renewable sources in conjunction with storage solutions. Moreover, seasonal energy dependency is largely overlooked in most existing studies. Greenhouses experience significant variations in energy demand throughout different seasons—during summer with higher sunlight and crop growth rates, and in winter when energy demands rise due to factors like lighting and heating needs. Understanding and optimizing these seasonal variations is critical for the design and management of energy systems to ensure long-term sustainability and efficiency. This study makes significant contributions to the field of sustainable agriculture and renewable energy integration.

1. This research focuses on enhancing energy autonomy in greenhouses equipped with STPV systems operating as microgrids, especially when integrated with BESS. By examining seasonal variations in energy autonomy and quantifying the impact of BESS on reducing reliance on the main grid, our study offers valuable insights into improving both energy efficiency and sustainability in greenhouse operations. Unlike prior works, which often lack a detailed exploration of seasonal dependency, this study provides a nuanced understanding of how BESS can be optimized to handle varying energy demands throughout the year.

2. The study introduces the use of the Harmony Search (HS) metaheuristic algorithm for optimizing the capacity and spatial distribution of BESS-STPV systems within the greenhouse environment. This novel approach enhances the ability to efficiently manage energy resources by considering dynamic system configurations that adapt to changing demand and seasonal factors. While existing research explores STPV and BESS independently, this study bridges the gap by integrating advanced optimization techniques that improve system performance and scalability.

3. Maintaining optimal conditions for crop growth is essential in greenhouse environments. By incorporating DLI constraints into the optimization model, the study ensures that energy solutions not only meet the greenhouse’s energy demands but also support healthy plant development. Unlike previous studies that prioritize energy efficiency alone, this approach highlights the importance of balancing energy production with agronomic needs, providing a holistic solution that addresses both energy and agricultural requirements.

The rest of the paper is organized as follows: section “Methodology” describes the study’s methodology in depth, providing a comprehensive overview of the approach used to investigate energy management strategies in greenhouse operations. Section “Mathematical formulation” outlines the objective functions, constraints, and the application of the HS algorithm for optimizing the integration of BESS with STPV systems. Section “Results” presents the findings, illustrating the outcomes of applying the optimized BESS-STPV configurations to a case study scenario during both winter and summer seasons, with comparisons made between scenarios with and without BESS. The Discussion in section “Results” interprets these findings. Section “Discussion: achievements and limitations” concludes the discussion and offers potential directions for further study in the area.

## Methodology

**Fig. 2:**
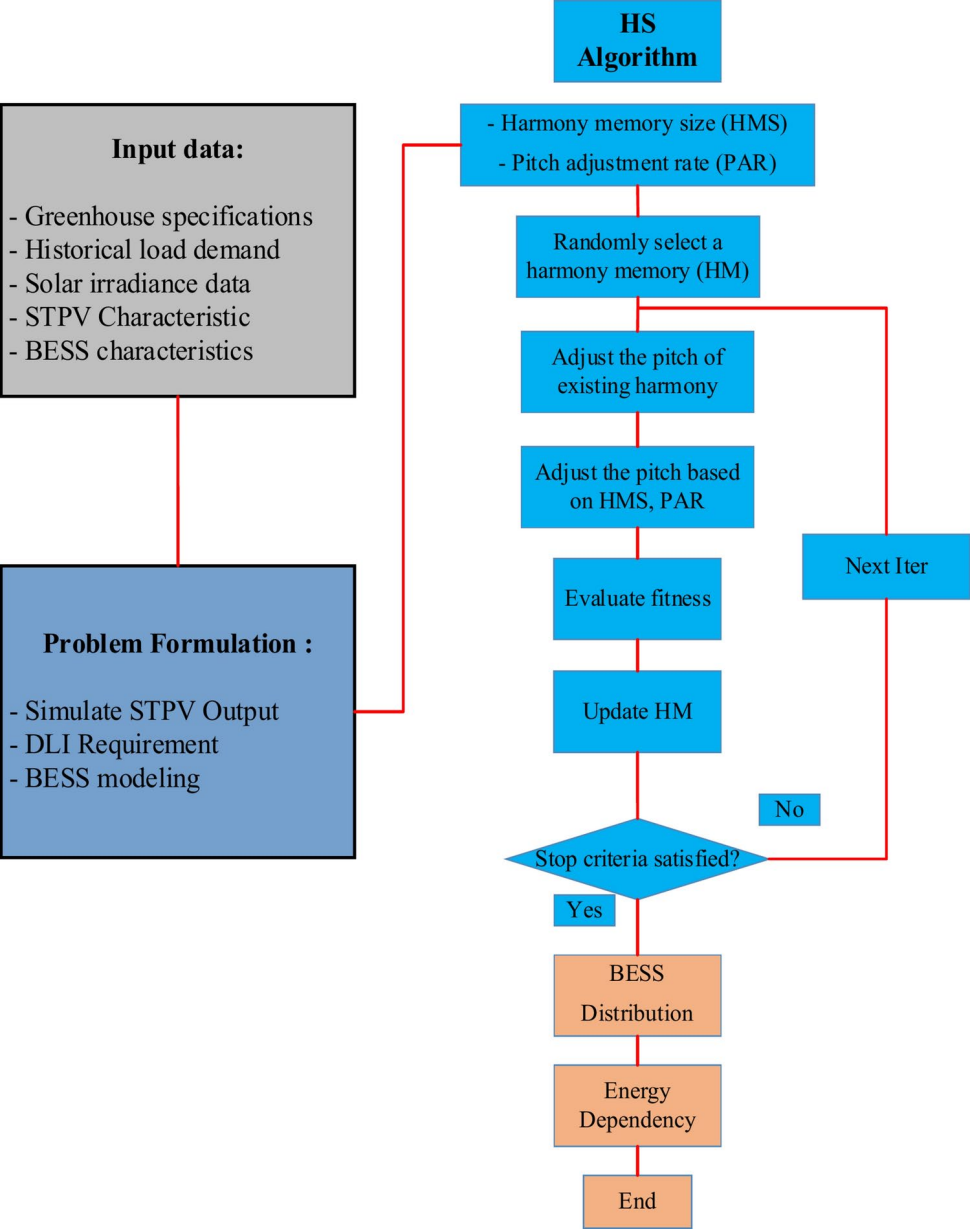
Flowchart of the proposed methodology.

In this section, we detail the approach used to investigate and optimize the integration of BESS with STPV systems in greenhouse operations. The proposed method’s flowchart is given in Fig. 2. First, input data pertinent to the study were collected and analyzed. This included greenhouse specifications, such as dimensions and structural characteristics, as well as load demand profiles for both summer and winter seasons. Additionally, data on solar irradiance and PV output specific to the location and orientation of the greenhouse were gathered to simulate energy generation scenarios under varying seasonal conditions. The characteristics of BESS, including storage capacity, efficiency, and associated costs, were also considered in the input data. To establish a baseline, the first stage is to compute the greenhouse’s energy autonomy without the integration of BESS. The ratio of imported electricity to the overall load demand is described as energy autonomy. Subsequently, HS is employed to find the optimum spatial distribution and capacity of BESS within the greenhouse. The Harmony Search (HS) algorithm was selected due to its unique advantages in optimizing the BESS-STPV system. Compared to other metaheuristic algorithms such as Genetic Algorithms (GA) or Particle Swarm Optimization (PSO), HS offers simplicity in implementation with fewer control parameters, making it easier to apply in practical scenarios. Additionally, HS excels in handling complex and nonlinear problems, which is essential for managing the interactions between renewable energy generation, energy storage, and crop growth. Unlike algorithms like GA, which may require extensive fine-tuning, HS effectively balances fast convergence with a lower likelihood of falling into local optima, ensuring robust and reliable results. This flexibility allows HS to adapt to the dynamic changes in seasonal energy availability and system requirements in greenhouse environments, providing a more efficient and tailored solution.

The objective was to minimize energy autonomy while ensuring that the DLI requirements for crop growth were met. DLI, a critical parameter influencing plant photosynthesis and growth, was integrated into the optimization framework as a constraint to maintain optimal growing conditions. Following the optimization process, energy autonomy calculations were repeated for scenarios incorporating the optimized BESS configurations during both summer and winter seasons. These calculations allowed for a comparative analysis, evaluating the effectiveness of BESS in reducing energy autonomy and enhancing energy autonomy in greenhouse operations under varying seasonal conditions.

The following is an explanation of the steps in the approach:

Step1: Input Data Collection:

- Gathered greenhouse specifications including dimensions, orientation, and structural details.
- Collected historical load demand data for both summer and winter seasons to characterize energy consumption patterns.
- Obtained solar irradiance data specific to the location and orientation of the greenhouse to simulate PV system output under varying seasonal conditions.
- Compiled characteristics of the BESS, including storage capacity, efficiency, and cost parameters.

Step2: Calculation of Initial Energy Autonomy:

- Defined and calculated the baseline energy autonomy of the greenhouse without the integration of BESS.
- ED is quantified, providing a benchmark for comparison.

Step3: Harmony Search (HS) Optimization:

- Applied the Harmony Search algorithm.
- Formulated objective functions to minimize energy autonomy while adhering to constraints, particularly the DLI requirements critical for crop growth.

Iteratively adjusted BESS configurations based on harmony memory and pitch adjustment mechanisms to converge on near-optimal solutions.

- Integration of DLI Constraint: Incorporated DLI constraints into the optimization model to ensure that energy solutions maintained adequate light levels necessary for optimal plant photosynthesis and growth.
- Adjusted BESS operation schedules and configurations to balance energy storage and discharge with fluctuating solar availability and crop lighting requirements.

**Fig. 3:**
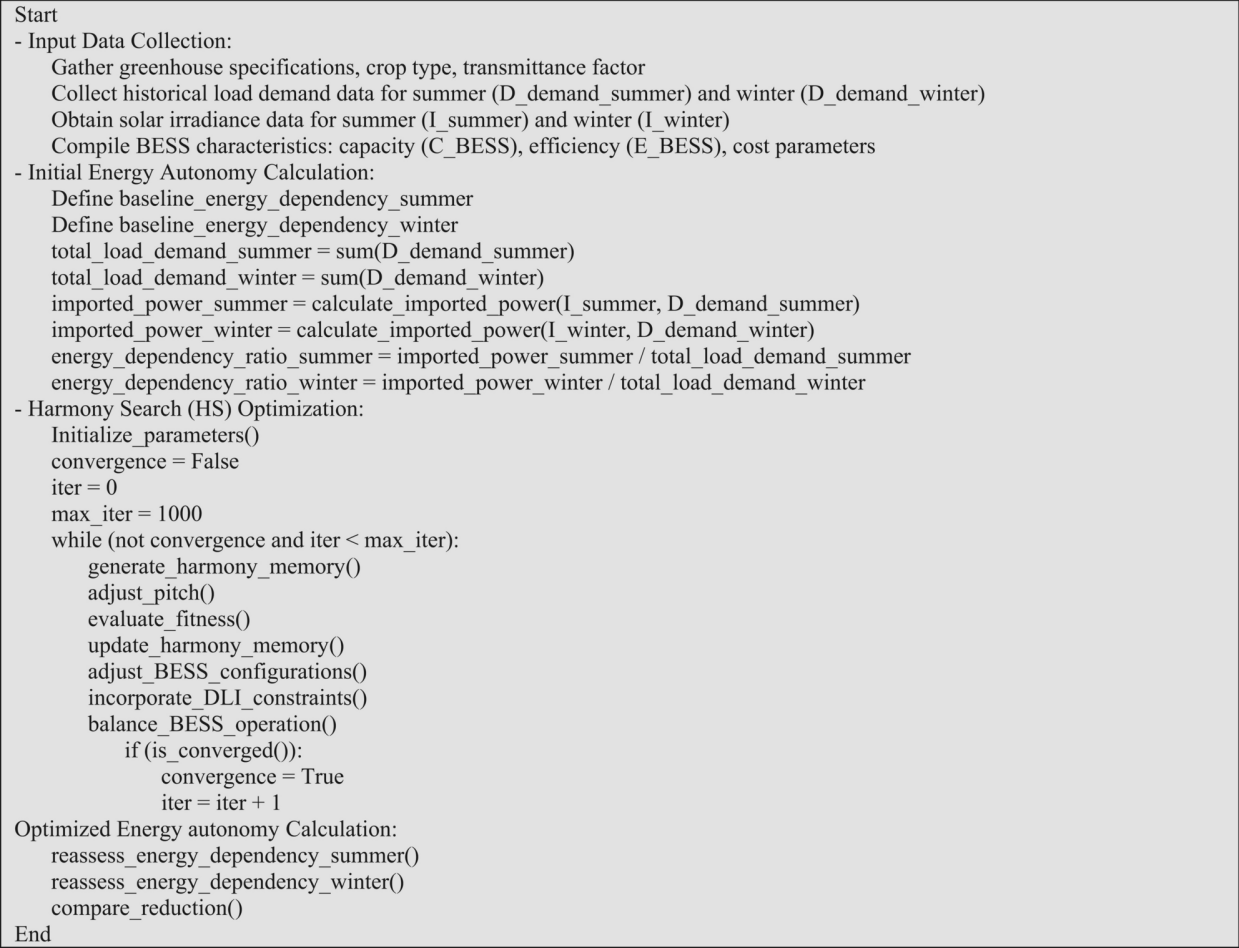
The pseudo code of the problem.

Step4: Calculation of Optimized Energy Autonomy:

- Reassessed energy autonomy calculations for scenarios incorporating the optimized BESS configurations during both summer and winter seasons.
- Compared and analyzed the reduction in energy autonomy achieved through BESS integration, evaluating its effectiveness in enhancing energy autonomy and sustainability in greenhouse operations. Furthermore, Fig. 3 provides the problem’s pseudo code.

## Mathematical formulation

This section details the mathematical formulation and optimization framework employed in the study. The formulation begins with defining an objective function aimed at minimizing the total cost associated with BESS, encompassing initial investment, operational expenses, and penalties for inadequate energy storage. Subsequently, the behavior of BESS is mathematically modeled, incorporating equations governing energy efficiency, charge–discharge cycles, and capacity constraints. To ensure optimal plant growth conditions, constraints based on the DLI are formulated, quantifying the minimum light intensity required by crops throughout the day. Energy autonomy, quantifying reliance on external power sources, is then calculated as a baseline metric. Finally, the HS algorithm is introduced to minimize energy autonomy by optimizing BESS operation, while meeting DLI constraints.

### The problem objective

The aim of the study is to minimize the total expenditure linked with the BESS. The study focuses on a greenhouse integrated with STPV panels and a BESS, operating as a microgrid for sustainable energy management. To simulate and optimize this system, a detailed modeling approach was implemented in MATLAB. This simulation environment enables a comprehensive analysis of energy generation, storage, and utilization within the greenhouse. In the simulation, key input parameters are considered, including STPV area, crop type, minimum DLI requirements, STPV system transmittance rates, and BESS capacity. Also, related cost parameters are fed into the problem. These factors are essential for accurately simulating the energy flow, allowing the assessment of STPV’s performance and the BESS’s role in optimizing energy autonomy and cost-effectiveness. The output of the simulation provides critical insights into the system’s energy performance, including energy autonomy, BESS utilization, and the effects of seasonal variations on energy management. By considering these factors, the simulation ensures that both energy efficiency and crop growth are maximized, offering a practical approach for managing renewable energy resources in greenhouse environments.

The objective function generally encompasses factors such as the initial capital outlay for BESS components, ongoing operational expenses (including maintenance and replacements), the cost of installation of the STPV, and expenses incurred from importing energy from the main grid (Eq. 1)^30^:

1 $$\min TC = C\_STPV + C\_BESS + C\_OPERATION + C\_GRID$$

Here, *TC* represents the total cost, $C\_STPV$ denotes the STPV installation cost, $C\_BESS$ refers to the cost associated with BESS, $C\_OM$ stands for the STPV-BESS system operational costs, and $C\_GRID$ represents the price of importing energy.

The price of BESS is determined using Eq. 2, represented by $Capacity_{BESS}$ and $Energy_{BESS}$:

2 $$C\_BESS = Capacity_{BESS} \times P\_rated + Energy_{BESS} \times E\_rated$$

Here, $P\_rated$ and $E\_rated$ denote the rated values of the BESS power and energy, respectively.

The operation ($OC_{STPV - BESS}$) and maintenance costs ($MC_{STPV - BESS}$) of STPV-BESS system is formulated by Eqs. 3 and 4:

3 $$OC_{STPV - BESS} = \sum\limits_{t = 1}^{NT} {CC(t)} + \frac{{RC_{STPV - BESS} }}{{LT_{STPV - BESS} }}$$

4 $$MC_{STPV - BESS} = \sum\limits_{t = 1}^{NT} {k_{cm} P_{STPV - BESS,t} }$$

where $CC(t)$ represents the charging cost of the system and $RC_{STPV - BESS}$ and $LT_{STPV - BESS}$ denote the substitute price and lifetime of the STPV-BESS, respectively. Additionally, $k_{cm}$ represents the maintenance cost coefficient per energy of the system. Also, *NT*, Is the total hours of the period.

The study assumes a three-tier time-of-use (ToU) pricing structure, with distinct rates for peak, intermediate, and off-peak hours. This pricing is determined based on the rates $\rho_{i,t}$ for peak, intermediate, and off-peak hours and related imported energy $IE_{t}$ (Eq. 5). This approach is consistent with existing energy policies and reflects realistic scenarios for agricultural energy consumers. ToU pricing captures the dynamic nature of electricity costs, making it a practical and widely recognized parameter for evaluating energy management strategies in microgrid systems.

5 $$C\_GRID = \sum\limits_{t = 1}^{NT} {\rho_{i,t} \times IE_{t} }$$

Moreover, the energy autonomy factor (*EAF*) is a vital indicator that’s utilized to assess the reliance of the greenhouse on external power sources. This factor serves as an indicator of how much the greenhouse depends on external energy supplies, which has implications for both cost and sustainability.

6 $$EAF = \frac{{\sum\limits_{t = 1}^{NT} {IE_{t} } }}{{\sum\limits_{t = 1}^{NT} {Load_{t} } }} \times 100$$

### BESS modeling

The BESS itself is modeled using Eqs. 7 to 11. The energy which can be stored in the BESS $E_{ESS,T}$, calculated based on the BESS power in charging/discharging process ($P_{ESS}^{c}$ and $P_{ESS}^{d}$), given in Eq. 7^16,31^.

7 $$E_{ESS,T} = E_{ESS,T - 1} + (P_{ESS}^{c} \eta_{c} - \frac{{P_{ESS}^{d} }}{{\eta_{d} }}) \times \Delta t\quad \forall t \in NT$$

Here, respective efficiencies of charge and discharge are presented by $\eta_{c}$ and $\eta_{d}$.

In the modeling of the BESS, we assume that the battery does not experience degradation during the analysis period. Battery degradation can be highly variable depending on factors like usage patterns, temperature, and charge–discharge cycles, making it challenging to generalize in initial analyses. Instead, only the cost of replacement is considered in the event of a battery’s operational lifespan ending. This simplification aligns with the common approach in preliminary feasibility studies, where degradation modeling is often excluded to focus on broader system performance metrics. By focusing solely on replacement costs, the model simplifies the calculation while still reflecting the financial impact of battery lifespan limitations.

Another important factor influencing the BESS’s performance is its state of charge, $SoC(t),$ which shows the amount of stored charge. The SoC factor is modeled by Eqs. 8 and 9, where stands for the maximum charge rate and for the minimum charge rate, respectively.

8 $$SoC_{\min } \le SoC(t) \le SoC_{\max } \quad$$

9 $$SoC(t) = SoC(t - 1) + \left\{ \begin{gathered} DC_{b} (t)\quad P(t) > 0 \hfill \\ CC_{b} (t)\quad P(t) > 0 \hfill \\ \end{gathered} \right\}$$

here, $DC_{b}$ and $CC_{b}$ are the discharge and charge consumed by battery respectively.

In addition, Eqs. 10 and 11 constrain the power $P_{ESS,t}$ and energy $E_{ESS,t}$ of the BESS within their rated values, determined by the BESS type^32^.

10 $$- P_{ESS}^{R} \le P_{ESS,t} \le P_{ESS}^{R} \quad \forall t \in NT$$

11 $$0 \le E_{ESS,t} \le E_{ESS}^{R} \quad \forall t \in NT$$

### Problem constraint

The load balance in the greenhouse is the primary constraint on the issue as a microgrid, minimum DLI based on the crop type, and the area of the STPV installed at the roof of the greenhouse, which are described below. First, as given in Eq. 12, the load demand at each hour should be met by STPV and BESS and imported power by the main grid.

12 $$P_{STPV} + P_{BESS} + P_{Grid} = Load_{t} \quad \forall t \in NT$$

It should be noticed that $P_{BESS}$ is considered a negative value when charging (considered as a load) in this formula.

Regarding the DLI, Photosynthetically Active Radiation (PAR) is an important factor necessary for the process of photosynthesis^26^. DLI is calculated based on the average sum of PAR for each crop in a day. Crops are categorized based on their light needs into high light-demanding (e.g., tomato, cucumber, sweet pepper), medium light-demanding (e.g., asparagus), and low light-demanding (e.g., certain floricultural crops) groups, requiring optimal DLIs of over 30, 10–20, and 5–10 mol/m^2^ d, respectively^27^.

The DLI is calculated using Eqs. 13 and 14 based on the average sum of outside and inside PAR received by the crop^26^;

13 $$SP = I_{O} \times f \times \alpha \times 0.0036$$

14 $$SP_{C} = SP \times \tau_{G}$$

In these equations, *SP* and *SP*_*c*_ represent the outside and inside PAR, respectively, both measured in the same units as DLI (mol/m^2^ d). The average daily irradiation is presented by $I_{O}$ in unit of (Wh/m^2^/d), and *f*, which is fixed at 0.48, is the ratio of PAR radiation to total solar radiation. A conversion factor of 0.0036 is used to change units from Wh/m^2^ to MJ/m^2^, and $\alpha$ is a coefficient set at 4.57 to change the unit of (MJ/m^2^) to the unit (mol/m^2^). Inside a greenhouse, the DLI, or daily light integral, is affected by the material used for the greenhouse roof. Typically, greenhouse roofs have high transmittance values,$\tau_{G}$, averaging around 0.9, though this can vary with different materials. In our study, we propose using STPV. The transmittance of these panels is lower than that of traditional greenhouse materials, which impacts our optimization analysis. The transmittance of panels is considered uniform across the panels, and their installation is limited to the greenhouse roof area. Uniform transmittance simplifies the model while focusing on system-level energy generation and crop lighting impacts. Area constraints reflect practical limitations in greenhouse design. By considering this factor, our study aims to optimize the use of STPV panels while ensuring that crops receive adequate light for growth. In this study, the minimum required DLI is another important constraint applied to make sure that the $SP_{C}$ inside the greenhouse should be more than the minimum lighting threshold required for the crop $LDI_{\min ,crop}$ (Eq. 15). This ensures that crops receive adequate light for photosynthesis and growth.

15 $$SPc_{crop} \ge LDI_{\min ,crop}$$

Finally, the total area used to install the STPV should not be more than the roof of the greenhouse (Eq. 16):

16 $$A_{STPV} \le A_{Greenhouse}$$

Moreover, Temperature plays a pivotal role in the efficiency of STPV panels, particularly in climates with extreme heat, such as Qatar. High temperatures can reduce the electrical efficiency of photovoltaic systems, thereby impacting energy autonomy. The efficiency of the STPV system at a given operating temperature can be expressed as:

17 $$\eta_{t = \;} \eta_{ref} \times \;\left( {1 - \beta \;\left( {T - T_{ref} } \right)} \right)$$

where $\eta_{t}$ is the efficiency of the STPV system at temperature t. $\eta_{ref}$ is the efficiency at the reference temperature $T_{ref}$ (typically 25 °C). Also, $\beta$ (typically −0.35%) represents the temperature coefficient of efficiency, which quantifies the efficiency reduction per degree increase in temperature, and the operating temperature is shown by *T*. The values for $T_{ref}$ and $\beta$ are sourced from manufacturer specifications to ensure practical relevance. In the case of high-temperature regions like Qatar, the operating temperature often exceeds the reference, leading to reduced efficiency. By incorporating this formulation, we can more accurately assess the system’s performance and devise strategies, such as improved cooling or hybrid energy systems, to mitigate temperature-related losses and ensure stable energy outputs.

### HS algorithm

Determining the optimize value of BESS and its distribution during a day to minimize the total cost of a greenhouse; is an NP-Hard type problem. One prominent method shown in previous researches is utilizing the metaheuristic algorithms. In this study we employed the HS algorithm. The improvisation process in music served as the inspiration for the HS algorithm^24^. Just as musicians seek a harmonious state in which all musical instruments are in tune, the HS algorithm seeks an optimal solution by iteratively improving a population of potential solutions^25^. HS algorithm offers significant advantages over other optimization techniques, making it particularly suitable for optimizing the size and distribution of BESS in greenhouses. One of the primary benefits is its simplicity and ease of implementation. Unlike traditional optimization methods that often require complex mathematical formulations or gradient information, HS is straightforward to set up and execute^33^. This characteristic is especially valuable in practical applications where the problem at hand involves multiple variables and constraints, such as energy management in greenhouses. Additionally, the HS algorithm excels in flexibility and global search capability^34^. This flexibility and its global search mechanism reduces the likelihood of falling in regional optima, a common challenge faced by algorithms like hill climbing. These features make HS particularly adept at addressing the complexities of optimizing BESS configurations, where balancing multiple factors is essential. Finally, the HS algorithm’s convergence efficiency and versatility are noteworthy. It often converges faster to an optimal solution compared to other metaheuristic methods. This efficiency is due to HS’s effective exploration and exploitation mechanisms.

The HS algorithm works by generateing new harmonies using two primary control parameters: the pitch adjustment rate (PAR) and the harmony memory size (HMS). The process is described here:

- Initialization:

- Initialize a population of N harmonies at random, where N > HMS.
- Apply an objective function to each harmony’s fitness evaluation.

- Improvisation:

- Continue until the predetermined end point is reached: a. Choose one of the three randomly selected processes below to create a new harmony:
- Randomly select a harmony from the existing population.
- Pitch-correct one or more components of an existing harmony.
- Pitch should be adjusted with a probability of 1—PAR while taking into account memory of the best harmonies discovered.
  18 $$H_{new} = H_{old} + BW \times r_{i}$$

where, $r_{i}$ is selected randomly in the interval [−1, 1] and BW represents the bandwidth of the pitch.

- Evaluation and Update:

As it mentioned before, the fitness function employed here is to optimize the TC (Eq. 18).

19 $$Fittness \, Function = \, \min \;TC$$

- Completion:

Repeat the procedure until the maximum number of iterations is achieved or the result converges to the ideal value.

## Results

**Fig. 4:**
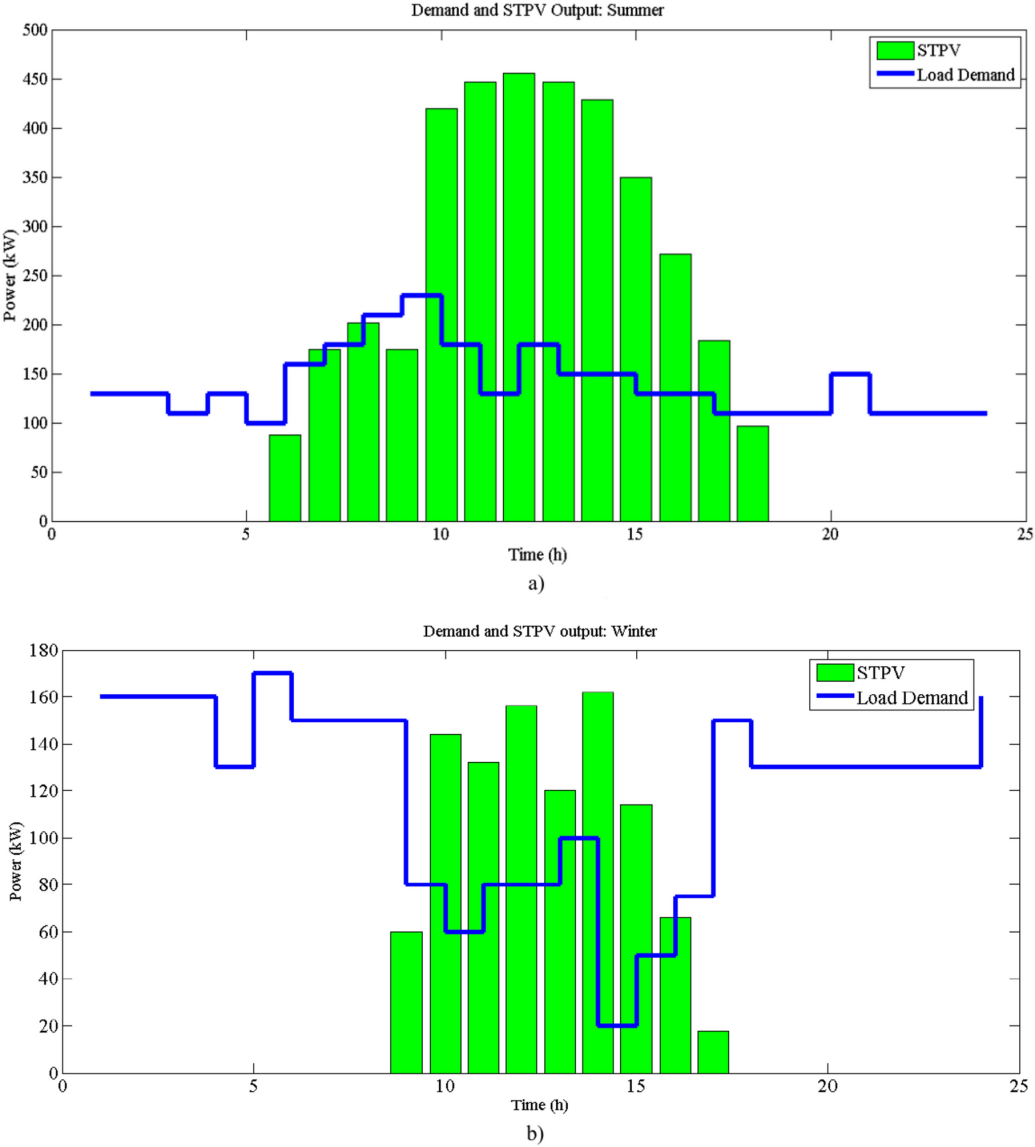
Load demand profile of the greenhouse and STPV output.

This section presents the outcomes of our study on optimizing the energy autonomy of a greenhouse equipped with STPV systems, with and without the integration of a BESS. We first detail the input data used for our simulations, including greenhouse specifications, load demands for both summer and winter, PV output for different seasons, BESS characteristics, and relevant cost parameters. Following this, we provide a comprehensive analysis of the results, considering the effect of BESS on energy autonomy during summer and winter. Additionally, the effectiveness of the HS algorithm in finding the optimum of BESS operation, while considering the DLI as a critical constraint, is evaluated and discussed. The greenhouse under study, as described in reference^35^, is a 24-acres building situated in the frigid environment of Essex County, Canada. Bell peppers are grown in this greenhouse, which has a 25-degree roof slope and a gutter height of 5.5 m. Figure 4a and b, respectively, depict the load demand profile and STPV output for a typical summer and winter day. In the referenced study, normal PV panels were utilized with a careful consideration of spacing , resulting in a ground surface to solar collector area ratio of 3^35^. In our study, we replace these normal PV panels with STPV panels. STPV panels, while having a lower efficiency compared to traditional PV panels, eliminate the need for spacing as they are integrated directly into the greenhouse structure. This allows us to use the entire greenhouse roof surface area for solar energy collection. To accurately adjust the PV output data from the reference study to reflect the characteristics of STPV panels, we applied an efficiency adjustment factor. This factor is based on the ratio of the efficiencies of STPV panels ($\eta_{STPV}$) to that of normal PV panels ($\eta_{PV}$). Additionally, we accounted for the full utilization of the greenhouse roof area, enhancing the total effective area available for solar energy harvesting. This adjustment ensures that the energy generation data used in our optimization process accurately represents the performance of STPV panels, considering both their lower efficiency and the increased area utilization afforded by their integration into the greenhouse structure.

The input data for our optimization problem includes various parameters related to electricity prices, BESS characteristics, and the HS algorithm. Table 3 summarizes these key data points. The electricity price is considered under a Time-of-Use policy. BESS characteristics include capacity, efficiency, and cost factors. Additionally, details about the HS algorithm, including its control parameters and settings, are provided.

**Table 3:** Input data for the optimization problem^36–38^.

Parameter	Value
Electricity Price(ToU)	Peak: 0.43 $ /kWh, Intermediate: 0.3 $ /kWh, Off-Peak: 0.12 $ /kWh
BESS Cost	$$P_{ESS}^{c}$$: 800 $ /kW and $$P_{ESS}^{d}$$: 0.37 $ /kWh
BESS Efficiency $$\eta_{c}$$ and $$\eta_{d}$$	0.8–0.9
BESS $$SoC_{\max } \quad$$, $$SoC_{\min }$$	0.2–0.8
HMS	20
PAR	30%
Number of Iteration	1000

**Fig. 5:**
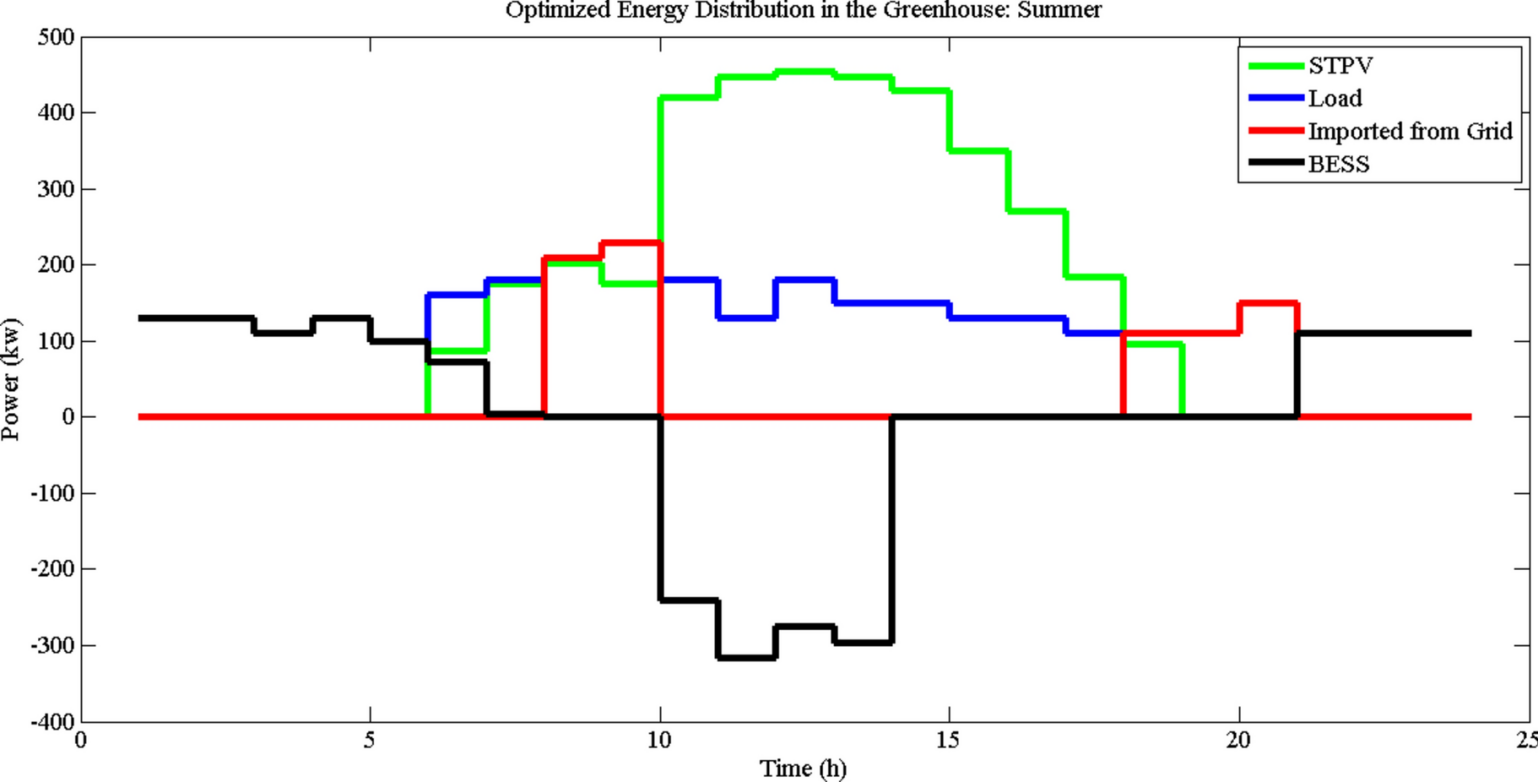
Energy distribution in the greenhouse in summer (LDI = 30, $\tau_{G}$ = 0.671).

**Fig. 6:**
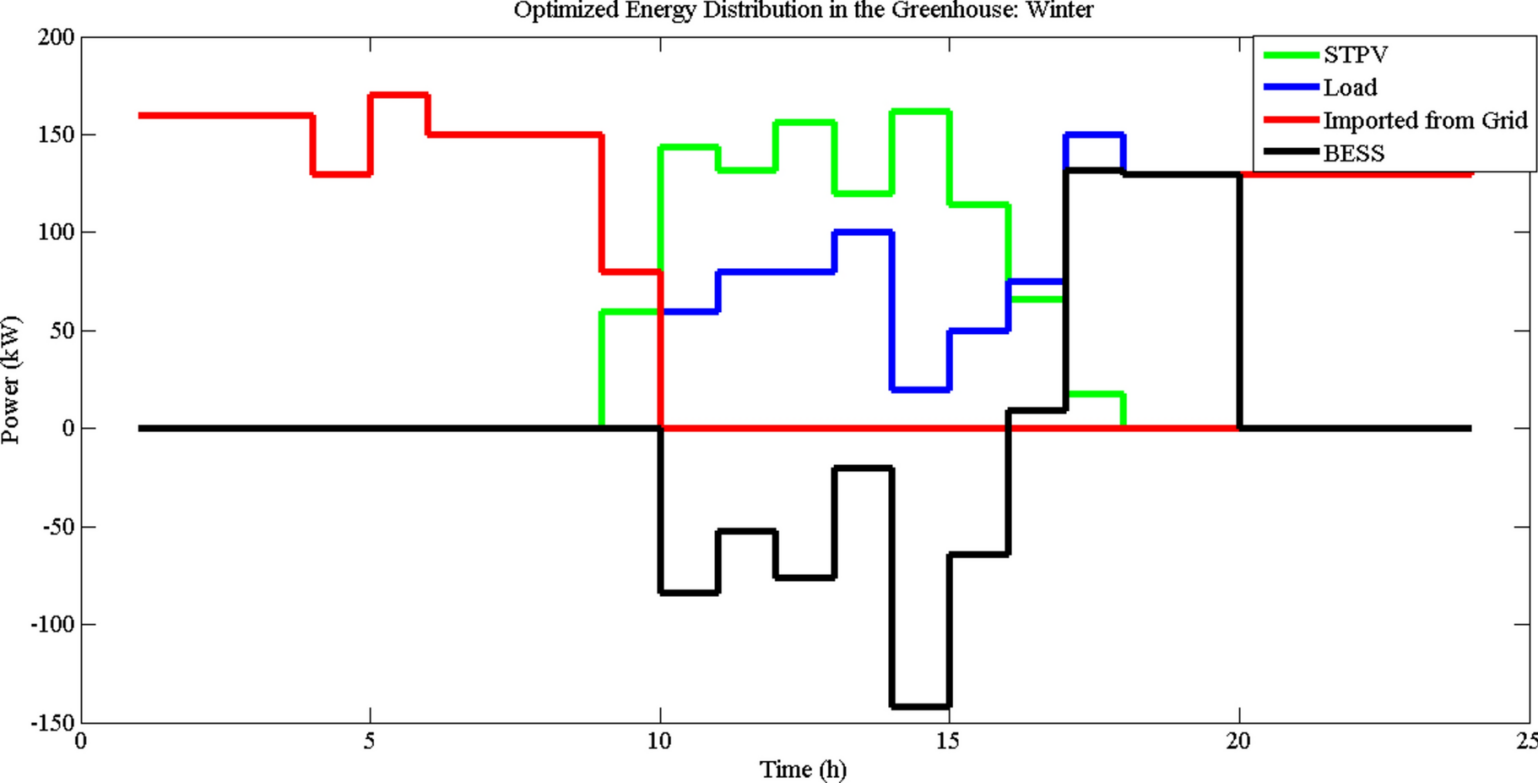
Energy distribution in the greenhouse in winter (LDI = 30, $\tau_{G}$ = 0.671).

Also, the transparency factor of the STPV panels significantly influences the amount of light transmitted into the greenhouse. We considered the transparency of the STPV panels, $\tau_{G},$ to be 0.671 and 0.597 as referenced from^26,28^. This value ensures that a substantial portion of sunlight can penetrate the panels, providing sufficient light for crop growth while simultaneously generating electricity. This transparency factor is incorporated into our optimization analysis to accurately reflect the dual functionality of the STPV panels. Using the HS algorithm, Figs. 5 and 6 depict the allocation of power within the greenhouse (LDI = 30, $\tau_{G}$ = 0.671), encompassing optimized distribution of the BESS, STPV, imported power from the grid, and load demand for summer and winter, respectively. As shown in the figures, the imported power during summer is less than in winter, and the utilization and impact of the BESS are greater in summer. In summer, the BESS charges between 10:00 and 14:00 when the STPV output is high, and discharges throughout the day to minimize energy imports. In winter, the BESS charges from 10:00 to 15:00 and discharges afterwards. However, during the morning and evening (after 20:00), the load must be supplied by the main grid due to the lower output of the STPV and insufficient excess power to charge the BESS.

Additionally, Table 4 illustrates the energy autonomy with and without BESS for both summer and winter.

**Table 4:** Calculated energy autonomy with and without BESS (LDI = 30, $\tau_{G}$ = 0.671).

Season	EAF-Without BESS	EAF-With BESS
Summer	43.43%	24.17%
Winter	81.36%	69.45%

The results of Table 4 indicate a significant reduction in energy autonomy when BESS is utilized. During summer, the energy autonomy decreases from 43.43% to 24.17%, showcasing the substantial impact of the BESS in managing energy needs efficiently. In winter, although the reduction is less pronounced, the energy autonomy still decreases from 81.36% to 69.45%. The higher reduction in energy autonomy during the summer can be attributed to the greater availability of STPV power, allowing the BESS to charge more effectively and thereby supply more power during periods of high demand. In contrast, the lower solar output during winter limits the effectiveness of the BESS, resulting in higher reliance on imported power from the grid. Nonetheless, the integration of BESS still provides a notable reduction in energy autonomy, demonstrating its importance in enhancing the energy resilience of greenhouses throughout the year.

Figures 5 and 6 illustrate the hourly energy distribution in the greenhouse during summer and winter, respectively, under the conditions of a minimum DLI requirement of 30 mol/m^2^/day and a transmittance value of 0.671. These figures show the contributions of STPV generation, BESS utilization, imported grid energy, and load demand over a 24-h period. In summer (Fig. 5), the optimization strategy suggests a more active role for BESS. During the early morning hours, stored energy from BESS is utilized to meet the load demand, eliminating the need for grid imports. This behavior highlights the importance of leveraging BESS to enhance energy autonomy during times of low solar generation. Additionally, during peak load hours later in the day, BESS is employed to reduce dependency on expensive imported energy, aligning with the time-of-use pricing policy. The increased utilization of BESS in summer is attributed to higher STPV generation during this season, which allows for greater energy storage and strategic discharge to minimize costs and reliance on external sources.

In contrast, Fig. 6 demonstrates a different energy distribution pattern in winter. Due to lower solar generation during this season, the optimization model often suggests that BESS remain inactive, with no significant charging or discharging occurring in the morning. Instead, energy is directly imported from the grid during this time to meet load demands. However, similar to summer, BESS is strategically utilized during peak load hours to reduce reliance on high-cost grid energy. This seasonal difference in BESS utilization reflects the impact of reduced solar availability in winter and the priority of minimizing operational costs through efficient energy management. Overall, the results highlight the seasonal dynamics of energy distribution in greenhouses. The higher STPV generation in summer allows for greater reliance on BESS, while the reduced generation in winter necessitates more direct grid imports. In both seasons, the optimization strategy prioritizes the use of BESS during peak load hours, aligning with economic considerations under ToU pricing. These findings underscore the importance of seasonal adjustments in energy management strategies to maximize efficiency and sustainability in greenhouse operations.

Furthermore, as previously noted, the minimum required DLI influences the selection of STPV and BESS, thereby affecting the energy autonomy in this study. Tables 5 and 6 illustrates the optimized BESS capacity and energy dependencies for various crop types and transmittance values for summer and winter respectively.

**Table 5:** Energy dependencies considering different required DLI and transmittance values: Summer.

Minimum DLI	$\tau_{G}$	STPV Allowed Area (m2)	BESS Capacity (kW)	EAF Without BESS (%)	EAF With BESS (%)
30	0.671	3237	330	43.43	24.17
	0.597	2596	260	47.81	27.12
20	0.671	3486	350	41.14	21.03
	0.597	3486	350	41.14	21.03
10	0.671	3486	350	41.14	21.03
	0.597	3486	350	41.14	21.03

**Table 6:** Energy dependencies considering different required DLI and transmittance values: Winter.

Minimum DLI	$\tau_{G}$	STPV Allowed Area (m^2^)	BESS Capacity (kW)	EAF Without BESS (%)	EAF With BESS (%)
30	0.671	3237	330	81.36	69.45
	0.597	2596	260	82.50	70.13
20	0.671	3486	350	79.15	64.51
	0.597	3486	350	79.15	64.51
10	0.671	3486	350	79.15	64.51
	0.597	3486	350	79.15	64.51

Tables 5 and 6 present the EAF for greenhouses during summer and winter, respectively, considering variations in the DLI, transmittance values, and BESS configurations. These tables highlight the impact of these factors on achieving energy autonomy and demonstrate the interplay between greenhouse energy demands, STPV areas, and BESS capacities under seasonal variations. In Table 5, the results for summer show that the DLI requirement significantly influences the allowed STPV area. For high DLI requirements, such as 30 mol/m^2^/day, the allowed STPV area decreases, especially at lower transmittance levels (e.g., 0.597). This reduction constrains energy generation and leads to higher EAF values both with and without BESS. Conversely, when DLI requirements are reduced to 20 or 10 mol/m^2^/day, the STPV area is maximized, enabling greater energy capture while adhering to the DLI constraints. Additionally, the role of BESS is pronounced in summer, where its integration significantly lowers the EAF. For instance, at a DLI of 30 mol/m^2^/day and a transmittance of 0.671, the EAF decreases from 43.43% to 24.17% with BESS, illustrating its critical role in reducing reliance on external power sources. Furthermore, higher transmittance values (0.671) enable larger STPV areas at high DLI requirements, thus improving energy generation and autonomy, whereas lower transmittance values (0.597) lead to stricter constraints and reduced autonomy.

Table 6 focuses on the winter scenario, where energy dependencies are generally higher due to reduced solar radiation. This seasonality is reflected in the higher EAF values compared to summer, even with the inclusion of BESS. For example, with a DLI of 20 mol/m^2^/day and a transmittance of 0.671, the EAF with BESS is 64.51%, indicating the increased challenge of achieving energy autonomy during winter. While BESS still contributes to lowering EAF in winter, the reduction is less significant compared to summer, emphasizing the need for robust storage and energy management solutions during low-irradiance seasons. Similar to summer, higher DLI requirements in winter constrain the STPV area and reduce energy autonomy. However, the effect of transmittance is more pronounced in winter, where lower transmittance values further exacerbate energy dependency. These findings underscore the importance of seasonal planning in greenhouse energy management. Achieving year-round energy autonomy requires dynamic adjustments to BESS and STPV configurations to accommodate varying DLI constraints and seasonal energy availability. Furthermore, the results highlight the need for tailored greenhouse designs that account for crop-specific DLI requirements, the transmittance properties of STPV panels, and the local energy dynamics across seasons.

Finally, Table 7 presents a detailed financial analysis of integrating a STPV system with a focus on cost components during summer and winter. The findings illustrate the economic benefits of such integration and its implications for energy management in greenhouse operations.

**Table 7:** Cost analysis with and without BESS Integration.

	Case	STPV Cost ($)	BESS Cost ($)	Imported from Grid ($)	Total Cost ($)	NPV after 20 Years ($)
Summer	Without BESS	90,000	75,000	76,277.7	241,277.7	712,392
	With BESS			43,740	208,740	946,730
Winter	Without BESS	90,000	75,000	125,251.92	290,251.92	373,980
	With BESS			107,460	272,460	415,813

In summer, the total operational cost of the greenhouse without a BESS is $241,277.7, with contributions from the STPV system ($90,000), fixed costs for the BESS infrastructure ($75,000), and energy imported from the grid ($76,277.7). However, when a BESS is integrated, the total cost reduces to $208,740, resulting in a cost saving of approximately $32,537.7 (13.5%). This reduction stems from the optimized utilization of the BESS. During off-peak hours, surplus energy from the STPV system is stored in the BESS, which is later discharged during peak demand hours. This strategic energy usage minimizes reliance on grid imports during high-cost periods. The lower BESS-related fixed cost ($43,740) further contributes to the overall cost reduction.

In winter, the total cost of operating the greenhouse without a BESS is $290,251.92, with the cost components being the STPV system ($90,000), BESS fixed costs ($75,000), and energy imported from the grid ($125,251.92). Integrating the BESS reduces the total cost to $272,460, yielding a cost saving of approximately $17,791.92 (6.1%). While the savings in winter are less pronounced compared to summer, the integration of the BESS remains beneficial. With lower solar energy generation during winter, the BESS is less utilized for storing surplus energy but is still employed effectively to reduce grid imports during high-cost periods. The ability to optimize energy consumption based on grid pricing demonstrates the versatility of the BESS in managing energy costs across varying seasonal conditions.

To evaluate the long-term economic feasibility of the proposed system, we calculated the Net Present Value (NPV) over 20 years, as presented in Table 7. The NPV calculations assume a 5% annual discount rate to account for the time value of money and a 3% annual increase in grid energy costs to reflect rising energy prices. A 20-year lifespan is assumed for the STPV system, with no significant replacement costs during this period. For the BESS, a 10-year lifespan is considered, requiring a single replacement cost at the 10-year mark. The analysis reveals that integrating BESS significantly enhances NPV in both summer and winter scenarios, with the greatest benefit observed in summer due to higher solar energy availability. Specifically, the NPV with BESS integration in summer is $946,730 compared to $712,392 without BESS, demonstrating its cost-effectiveness. In winter, the results show a modest improvement, with NPV increasing from $373,980 without BESS to $415,813 with BESS. This highlights the need for complementary renewable energy solutions, such as wind or biomass systems, to further reduce grid dependency during periods of lower solar output. Additionally, to further enhance system performance and economic feasibility, hybrid energy storage solutions such as hydrogen energy storage could be integrated. Hydrogen storage systems have the advantage of long-term energy retention and can address the seasonal variability of solar energy availability, particularly during winter months. By converting surplus solar energy into hydrogen through electrolysis and storing it for later use, greenhouses could significantly reduce grid dependency and improve the overall sustainability of the project.

Furthermore, Table 8 illustrates the EAF of the STPV-BESS system with and without considering the impact of temperature, highlighting the efficiency adjustments under various DLI levels and seasonal conditions.

**Table 8:** Energy dependencies in STPV-BESS scenario considering temperature impact.

Minimum DLI	Season	EAF without considering temperature	EAF With considering temperature	Increscent in EAF (%)
30	Summer	24.17	25.57	5.8
	Winter	69.45	70.63	1.7
20	Summer	21.03	22.31	6.1
	Winter	64.51	66.12	2.5
10	Summer	21.03	22.46	6.7
	Winter	64.51	66.18	2.6

When accounting for temperature effects ($T_{ref}$ = 25C, $\beta$ =  − 0.35%, and $\tau_{G}$ = 0.671), the results show a consistent improvement in EAF across all scenarios. For summer conditions with a DLI of 30 (mol/m^2^ d), EAF increased by 5.8%, from 24.17% to 25.57%. Similarly, winter conditions at the same DLI saw a 1.7% increment in EAF, rising from 69.45% to 70.63%. As the DLI requirement decreased to 20 and 10 mol/m^2^ d, the improvements were even more pronounced in summer, with increments of 6.1% and 6.7%, respectively. In winter, EAF rose by 2.5% and 2.6% for the same scenarios. These findings underscore the significance of incorporating temperature effects into energy management models, particularly in climates with high ambient temperatures.

Finally, the performance of the HS algorithm was benchmarked against Genetic Algorithm (GA) and Particle Swarm Optimization (PSO) for the STPV-BESS system under specific conditions (DLI 30 mol/m^2^ d, transmittance 0.671). The results, summarized in Table 9, highlight the key differences in EAF, convergence speed, and ease of implementation among these methods.

**Table 9:** Comparative evaluation of optimization algorithms for STPV-BESS system – DLI 30, T 0.671.

Criterion		HS	GA	PSO
EAF	Summer	24.17	27.13	24.76
	Winter	69.45	73.25	70.18
Convergence Speed (S)		1200	1500	1100
Ease of Implementation		Simple	Moderate	Moderate

In terms of EAF, HS outperformed GA and PSO slightly. However, the differences in EAF were relatively minor, indicating that all three methods provide comparable performance in optimizing energy autonomy. Regarding convergence speed, HS exhibited a significant advantage over GA, with a runtime of 1200 s compared to 1500 s for GA, demonstrating its efficiency in reaching optimal solutions. While PSO showed the fastest convergence (1100 s), it presented higher variability in EAF across multiple runs, indicating potential instability in certain scenarios. Lastly, the ease of implementation was rated highest for HS, owing to its straightforward structure and fewer parameters to tune, making it a practical choice for this study. Both GA and PSO were rated as moderate due to their more complex configurations and parameter dependencies. These findings underscore the balance HS offers better computational efficiency and practical implementation, justifying its selection as the primary optimization method for this research.

## Discussion: achievements and limitations

In this section, the key findings of the study are explained. We examine the effectiveness of integrating STPV panels and BESS in greenhouses, specifically focusing on the reduction of energy autonomy and carbon footprint. Additionally, the impact of various factors such as the minimum DLI and STPV transmittance on system performance is explored. Finally, the limitations encountered during the study to improve the integration of renewable energy solutions in agricultural practices are discussed. The findings of this study contribute to broader goals such as achieving carbon neutrality and enhancing energy security within the agricultural sector. By optimizing BESS and STPV systems, the research supports the transition towards more sustainable and self-sufficient agricultural practices. Carbon neutrality is increasingly emphasized in agricultural policies, where reducing dependence on fossil fuels and integrating renewable energy sources are key strategies. Through the efficient use of BESS-STPV systems, this study demonstrates how agricultural operations can move towards achieving net-zero carbon emissions, aligning with global efforts to combat climate change. Additionally, the study addresses energy security by ensuring that agricultural operations have a resilient and reliable energy supply, minimizing vulnerability to disruptions in grid electricity. These advancements contribute directly to fostering a more sustainable, secure, and environmentally responsible agricultural sector.

To enhance the practical relevance of the proposed STPV-BESS system, we compared our findings with two real-world studies that utilized similar approaches. The first study conducted in Greece (latitude 39.07°N) evaluated the energy generation capacity of greenhouses with STPV panels covering 50% and 100% of the roof area^39^. Case 1, with 500 m^2^ of coverage, achieved 63,750 kWh annually, meeting 80% of the greenhouse’s energy needs. Case 2, with 1,000 m^2^ coverage, generated 234,000 kWh annually, covering 100% of energy needs and enabling surplus energy to be exported to the grid. The second study conducted in Arizona, USA (latitude 32.25°N) reported that 49% coverage of the greenhouse roof with STPV was sufficient to meet the energy demands, highlighting the system’s viability even in different climatic conditions^40^. These comparisons illustrate the versatility and scalability of STPV systems for greenhouse energy autonomy, aligning well with our findings. Our study complements these findings by presenting a detailed analysis of energy autonomy improvements achieved through the integration of BESS with STPV systems. As shown in Table 4, in summer, the energy dependency without BESS was 43.43%. Similarly, in winter, the ED reduced from 81.36% without BESS to 69.45% with BESS. These results align with the aforementioned studies and highlight the ability of STPV-BESS systems to adapt to seasonal variations in energy demand while reducing reliance on grid electricity.

### Achievements

The findings underscore several achievements in optimizing the energy autonomy of greenhouses using STPV systems combined with BESS. These achievements are illustrated with numerical examples drawn from our data tables:

- Reduction in energy autonomy using BESS:

In summer, the implementation of BESS reduced energy autonomy from 43.43% to 24.17%, a substantial decrease of approximately 44%. Also, in winter, although the reduction was less pronounced, energy autonomy still decreased from 81.36% to 69.45%, indicating a 15% improvement.

- Effectiveness of BESS in different seasons:

The results highlight the variability in BESS effectiveness across seasons. In summer, the high output of STPV systems allowed for more effective BESS usage, resulting in a more significant reduction in energy autonomy. In winter, despite the lower STPV output and reduced charging opportunities for BESS, the system still contributed to a notable reduction in energy autonomy.

- Impact of STPV transmittance and minimum DLI on system performance:

The transmittance rate of STPV panels and the minimum required DLI for crops significantly influenced the system’s performance. For instance, with a minimum DLI of 30 mol/m^2^ d and a transmittance rate of 0.671, the energy autonomy in summer with BESS was 24.17%. However, with a reduced transmittance rate of 0.597, the energy autonomy increased to 27.12%. Similarly, in winter, with a DLI of 30 mol/m^2^ d and a transmittance rate of 0.671, the energy autonomy with BESS was 69.45%. This increased slightly to 70.13% with a transmittance rate of 0.597.

- DLI’s Role in optimizing STPV and BESS:

Lowering the DLI requirement had a noteworthy effect on system optimization. For the DLI 20 mol/m^2^ d, the energy autonomy in summer with BESS was 21.03%, irrespective of the transmittance rate, indicating that reducing DLI can facilitate better optimization of STPV and BESS capacity. In winter, the same DLI reduction led to a dependency of 64.51% with BESS, showing a consistent pattern of reduced energy autonomy with lower DLI requirements. These achievements demonstrate the potential of combining STPV systems with BESS to significantly reduce energy autonomy in greenhouses. However, they also highlight the critical roles that seasonal variations, transmittance rates, and minimum DLI requirements play in optimizing these systems. Despite the notable improvements, the energy autonomy in winter remains relatively high, indicating areas where further technologies and solutions are needed.

### Limitations

The discussion in the manuscript touches upon various aspects of integrating a Semi-Transparent Photovoltaic (STPV) system and Battery Energy Storage System (BESS), but there is a need to explore the practical limitations more comprehensively.

- High Initial Costs: One of the significant limitations is the high initial investment required for the implementation of STPV and BESS systems. While the cost-saving benefits are evident in the long term, the upfront expenses associated with the installation, maintenance, and infrastructure development can deter some greenhouse operators. These costs include the procurement of STPV panels, BESS infrastructure, and the necessary technological components for integration. Addressing this challenge requires a robust financial model and potential subsidies or incentives for sustainable energy solutions.
- Weather Condition Effects on Panel Efficiency and Output: Another critical limitation is the impact of weather conditions on STPV panel efficiency and energy output. Solar panels, including STPV systems, are highly sensitive to changes in weather, particularly cloud cover, temperature variations, and seasonal shifts. In regions with fluctuating weather patterns, the efficiency of STPV systems may decline, affecting energy generation and consequently, the effectiveness of the integrated BESS. This necessitates the development of adaptive solutions to optimize performance, such as dynamic control strategies or hybrid energy sources to complement solar power during low-yield periods.
- Technical Challenges in Implementing HS Optimization: The implementation of the Harmony Search (HS) algorithm for optimizing BESS performance also presents technical challenges. Despite its efficiency, HS may face difficulties in handling complex optimization problems with a high number of variables and constraints. Moreover, ensuring convergence to optimal solutions in a timely manner can be challenging, especially in dynamic operational environments like greenhouses where energy demand and environmental factors continuously change. Further research into refining HS or integrating it with other optimization methods can help mitigate these limitations.

Furthermore, future advancements in STPV and BESS have the potential to significantly address the observed challenges. Emerging technologies, such as more efficient solar cell designs for STPV systems, could improve energy conversion rates and increase the area’s power generation capacity. Advances in energy storage, such as the development of solid-state batteries or flow batteries, could enhance BESS performance by providing higher energy density, faster charge/discharge cycles, and longer lifespans. Additionally, the integration of artificial intelligence (AI) and machine learning into optimization frameworks could optimize BESS operations more dynamically, allowing for real-time adjustments based on weather patterns, energy demand fluctuations, and grid interactions. These technological advancements would further improve the efficiency, sustainability, and reliability of the overall system, addressing key challenges related to energy management, cost-effectiveness, and resilience in agricultural microgrids.

## Conclusion

This paper underscores the critical importance of integrating renewable energy solutions into greenhouse operations to enhance sustainability and reduce energy autonomy. The integration of STPV systems and BESS presented a promising approach to achieving these goals. The methodology involved a detailed examination of a greenhouse in Essex County, Ontario, Canada, producing bell peppers. By using the HS algorithm, we optimized the size and distribution of the BESS while considering the DLI requirements for different crops as a primary constraint. The study aimed to minimize the total cost associated with the BESS and STPV system while ensuring adequate light levels for crop growth.

To implement the proposed system in real-world settings, it is essential to consider practical challenges such as cost, technical feasibility, and seasonal variability. Future research directions could explore alternative STPV materials that enhance efficiency in low-light conditions and develop dynamic DLI management strategies that adapt to changing environmental factors. Additionally, integration of hybrid systems combining multiple renewable energy sources, such as wind, biomass, or geothermal energy, could further optimize energy storage and usage in greenhouses.

The following highlights this study’s major outcomes: Firstly, the implementation of BESS significantly reduced EAF. For instance, in summer, the EAF decreased from 43.43% without BESS to 24.17% with BESS. Similarly, in winter, the EAF decreased from 81.36% without BESS to 69.45% with BESS, showcasing the effectiveness of BESS in lowering reliance on grid power. Secondly, the study demonstrated that the transmittance rate of STPV panels and the minimum required DLI are crucial factors in optimizing the energy system. By optimizing these factors, the system effectively balances energy generation and crop lighting needs, ensuring more efficient use of renewable energy resources. For instance, in summer, reducing the transmittance rate from 0.671 to 0.597 improved energy autonomy from 24.17% to 27.12%, while in winter, lowering DLI from 30 mol/m^2^ d to 20 mol/m^2^ d maintained energy autonomy at 64.51% with BESS. These findings emphasize the importance of tailoring STPV and BESS systems to crop-specific lighting demands, enhancing both sustainability and energy resilience. Furthermore, incorporating strategies for dynamic DLI management and the integration of complementary renewable energy sources, such as wind or biomass, will further optimize system performance across varying seasonal conditions. Thirdly, seasonal variations significantly impacted energy autonomy. In summer, the STPV output was sufficient to charge the BESS effectively, resulting in lower energy autonomy. However, in winter, due to lower solar radiation, the effectiveness of BESS was diminished, highlighting the need for additional optimization or supplemental renewable sources during the winter months.

To enhance the applicability of these findings, actionable insights are provided for researchers, policymakers, and practitioners. Researchers are encouraged to further explore the integration of STPV and BESS systems in diverse agricultural contexts, focusing on optimizing the balance between energy generation and crop lighting needs. Policymakers can support this integration by creating supportive policies and financial incentives to promote sustainable agricultural practices. Practitioners, such as greenhouse operators, can utilize these insights to enhance energy management, reduce operational costs, and improve the sustainability of their operations. Also, to address seasonal challenges, future work should focus on exploring advanced energy storage solutions and integration of supplementary renewable energy sources such as wind or biogas systems to improve BESS performance in winter months. Additionally, dynamic DLI management strategies can be developed to adapt to seasonal variations, ensuring that crops receive optimal light for growth throughout the year. By addressing these achievements, this research provides insightful information about the optimization of renewable energy systems in greenhouses. The integration of STPV and BESS not only enhances energy sustainability but also supports the operational needs of greenhouses, ensuring reliable and efficient energy usage throughout the year. Future research should further explore the variability of STPV technologies, regional differences, and detailed economic analyses to strengthen the applicability and robustness of these renewable energy solutions.

## Data Availability

All required data are included in the manuscript. Additional data can be made available upon request by contacting Dr. S M Muyeen at (sm.muyeen@qu.edu.qa) or Mohammadreza Gholami at (mohammadreza.gholami@final.edu.tr).
